# Supplementary material for: Complex‐centric proteome profiling by SEC‐SWATH‐MS
Source: Mol Syst Biol. 2019 Jan 14;15(1):e8438. doi: 10.15252/msb.20188438 (PMC6346213; doi:10.15252/msb.20188438)
Supplement: Supplementary file 8 — Dataset EV7 [file MSB-15-e8438-s008.zip › feature_plots_string/P30613.pdf]

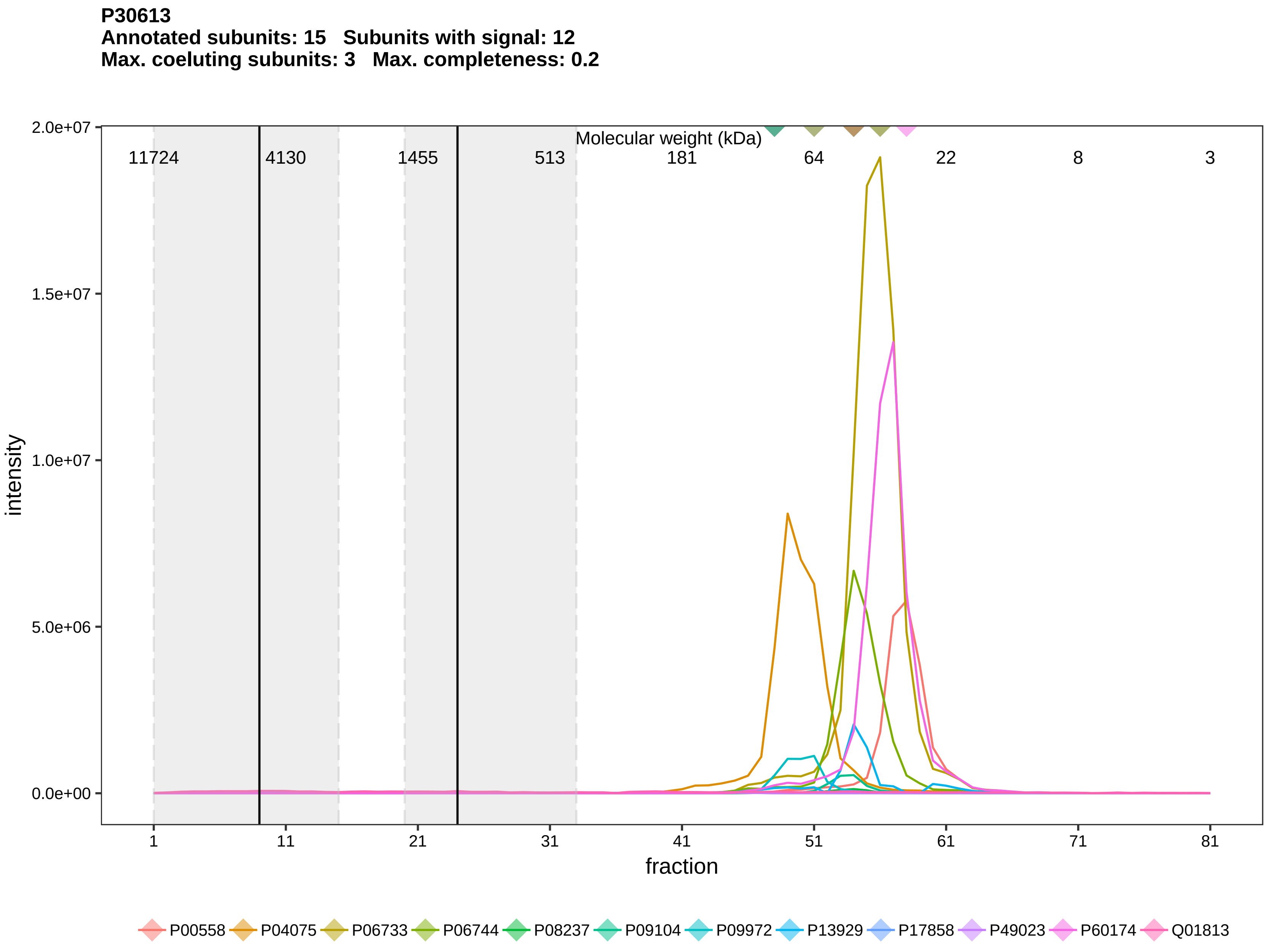

P30613
Annotated subunits: 15 Subunits with signal: 12
Max. coeluting subunits: 3 Max. completeness: 0.2
Molecular weight (kDa)
11724
4130
1455
513
181
64
22
8
3
2.0e+07
1.5e+07
1.0e+07
5.0e+06
0.0e+00
intensity
1
11
21
31
41
51
61
71
81
fraction
P00558
P04075
P06733
P06744
P08237
P09104
P09972
P13929
P17858
P49023
P60174
Q01813
